# Acetyl-L-Carnitine in Dementia and Other Cognitive Disorders: A Critical Update

**DOI:** 10.3390/nu12051389

**Published:** 2020-05-12

**Authors:** Manuela Pennisi, Giuseppe Lanza, Mariagiovanna Cantone, Emanuele D’Amico, Francesco Fisicaro, Valentina Puglisi, Luisa Vinciguerra, Rita Bella, Enzo Vicari, Giulia Malaguarnera

**Affiliations:** 1 Department of Biomedical and Biotechnological Science, University of Catania, Via Santa Sofia 89, 95123 Catania, Italy; manuela.pennisi@unict.it (M.P.); drfrancescofisicaro@gmail.com (F.F.); giulia.malaguarnera@live.it (G.M.); 2 Department of Surgery and Medical-Surgical Specialties, University of Catania, Via Santa Sofia 78, 95123 Catania, Italy; 3 Department of Neurology IC, Oasi Research Institute-IRCCS, Via Conte Ruggero 73, 94018 Troina, Italy; 4 Department of Neurology, Sant’Elia Hospital, Azienda Sanitaria Provinciale (ASP) Caltanissetta, Via Luigi Russo 6, 93100 Caltanissetta, Italy; m.cantone@asp.cl.it; 5 Department of Medical and Surgical Sciences and Advanced Technologies, University of Catania, Via Santa Sofia 78, 95123 Catania, Italy; emanuele.damico@unict.it (E.D.); rbella@unict.it (R.B.); 6 Department of Neurology, Azienda Socio-Sanitaria Territoriale (ASST) Cremona, Viale Concordia 1, 26100 Cremona, Italy; valentina.puglisi@asst-cremona.it (V.P.); luisa.vinciguerra@asst-cremona.it (L.V.); 7 Department of Clinical and Experimental Medicine, University of Catania, Via Santa Sofia 89, 95123 Catania, Italy; enzodante@email.it; 8 Research Center “The Great Senescence”, University of Catania, Via Androne 83, 95124 Catania, Italy

**Keywords:** acetyl-L-carnitine, neurodegeneration, dementia, mild cognitive impairment, memory loss, biochemistry, neuroplasticity, hepatic encephalopathy, gut–liver–brain axis

## Abstract

Several studies explored the effects of acetyl-L-carnitine (ALC) in dementia, suggesting a role in slowing down cognitive decline. Nevertheless, in 2003 a systematic review concluded there was insufficient evidence to recommend a clinical use, although a meta-analysis in the same year showed a significant advantage for ALC for clinical scales and psychometric tests. Since then, other studies have been published; however, a critical review is still lacking. We provide an update of the studies on ALC in primary and secondary dementia, highlighting the current limitations and translational implications. Overall, the role of ALC in dementia is still under debate. The underlying mechanisms may include restoring of cell membranes and synaptic functioning, enhancing cholinergic activity, promoting mitochondrial energy metabolism, protecting against toxins, and exerting neurotrophic effects. The effects of ALC on the gut–liver–brain axis seem to identify the category of patients in which the new insights contribute most to the mechanisms of action of ALC, likely being the liver metabolism and the improvement of hepatic detoxifying mechanisms the primary targets. In this framework, our research group has dealt with this topic, focusing on the ALC-related cross-talk mechanisms. Further studies with homogeneous sample and longitudinal assessment are needed before a systematic clinical application.

## 1. Introduction

### 1.1. Background

Dementia is a common and disabling neurological disorder worldwide. The World Alzheimer Report of 2015 underlined that over forty-six million individuals across the globe have dementia, with the number expected to rise to over seventy-four million by 2030 and a further increase to over a hundred and thirty-one million individuals by 2050 [1]. Typical clinical features include an acquired impairment of cognitive functions and changes in mood, behavior, and personality, eventually leading to loss of functional independence. Neuropathological findings highlight differences in the existing types of dementia, although, clinically, memory impairment is observed in most cases and has been linked with the degeneration in cholinergic neurons and reduced levels of brain choline acetyltransferase [2]. Other findings are a widespread loss of neuronal cells and synaptic dysfunction, which might be the result of free radical release and abnormal energy processes.

The possibility for prevention and treatment of Alzheimer’s disease (AD), which is the most fearsome and frequent type of dementia, is one of the challenges of modern research. Studies on the early stages of AD have led to the identification and characterization of a transition state between normal brain aging and AD, known as Mild Cognitive Impairment (MCI) [3]. In MCI, the affected individuals experience symptoms, such as memory loss and/or other cognitive deficits, that are more severe than those expected for age and educational level but do not satisfy the diagnostic criteria for dementia. Individuals with MCI have a higher risk for developing AD, which makes it an ideal scenario for the development of strategies for prevention and intervention [4].

A causative treatment for AD and other degenerative dementias is yet to be discovered, with the available treatment options offering only symptomatic benefits [5,6,7]. To date, the medications approved for the treatment of AD include the acetylcholinesterase (AChE) inhibitors (tacrine, donepezil, rivastigmine, and galantamine) and the memantine, which is a N-methyl-D-aspartate (NMDA) glutamate receptor antagonist. However, tolerance and compliance to these drugs are variable and studies showed that they cannot delay or halt the disease progression [8]. Recently, some non-pharmacological strategies seem to be encouraging, although a definitive conclusion on their efficacy and long-lasting effects cannot be drawn yet [6,7,9,10,11,12,13,14].

### 1.2. Acetyl-L-Carnitine

Acetyl-L-carnitine (ALC) is an ester of the trimethylated amino acid L-carnitine and it is synthesized in the human brain, liver, and kidney by the enzyme ALC-transferase [15]. ALC facilitates the uptake of acetyl-CoA into the mitochondria during fatty acid oxidation, enhances the acetylcholine production, and stimulates the synthesis of proteins and membrane phospholipids [16,17].

L-carnitine and ALC can be administered orally, intravenously (IV), or intramuscularly. They are absorbed in the jejunum by simple diffusion and transported into cellular tissue through an active transport mechanism, with plasma concentrations reaching an equilibrium via the carnitine acetyl-transferase activity [18]. Both IV and oral administration results in a corresponding increase in cerebrospinal fluid (CSF) concentrations of ALC, indicating that it readily crosses the blood-brain barrier (BBB). In a previous study on healthy fasting men, a single 500 mg dose of ALC yielded a maximal plasma concentration of 1.19 μg/mL at 3.1 h post-dose; ALC half-life was 4.2 h, with an area under the curve of 9.88 μg·h/mL [19]. L-carnitine and its esters undergo minimal metabolism and are subsequently excreted in the urine via the renal tubular reabsorption, with a rate of clearance that increases with their plasma concentration [20].

Although the exact mechanism of action of ALC is currently unknown, studies indicate that it may be related to its activity on cholinergic neural transmission and ability to enhance neuronal metabolism in the mitochondria [21]. Some researchers attribute the cholinergic effects of ALC to the blocking of post-synaptic inhibition potentials, whereas others suggest a direct synaptic stimulation [22,23]. Based on the enhanced cellular energetics in the mitochondria, human studies show that ALC has the property to stabilize the cell membrane fluidity through the regulation of sphingomyelin levels, and to provide a substrate reservoir for cellular energy production, thereby preventing excessive neuronal degeneration [24]. ALC has also been shown to increase the hippocampal binding of glucocorticoids and nerve growth factor [25], to reduce the oxidative stress, and to inhibit the excitotoxicity in brain tissue and CSF, thus preventing cell death and ischemia-induced neuronal damage [26,27]. Regarding the effects on the peripheral nervous system, the benefits of ALC supplementation in patients with peripheral neuropathies may be attributed to its role in nerve regeneration and protection, as well as to its antioxidant, antiapoptotic, and analgesic activity [28].

### 1.3. ALC And Dementia

Based on preclinical and laboratory observations, ALC has been translated in human clinical research as an adjuvant treatment in patients with dementia or MCI [16,29], geriatric depression [30,31,32], chronic fatigue syndrome, Multiple Sclerosis-related fatigue [33], autism spectrum disorders [34], as well as different types of peripheral neuropathies [35] and other common medical conditions.

In cognitive disorders, the research has been focusing on exploring the effects of ALC since the 1980s, although the studies were relatively small, uncontrolled, and often unpublished. However, the initial findings were encouraging and stimulated further controlled trials that contributed to the role of ALC in enhancing cognitive functions in normal elderly and in ameliorating cognitive impairment in patients with AD or MCI. Nevertheless, the inconclusive results of a larger study indicated that those benefits were not substantial [36]. Furthermore, a 2003 Cochrane systematic review including 16 trials concluded that, even though some benefits of ALC were evident on the clinical global impression (CGI) and the Mini Mental State Examination (MMSE) at 24 weeks, there was little-to-no evidence when it came to employing objective assessments in other outcome measures [37]. The other aspect noted was that a large number of these studies had vast differences in the methodology and assessment tools employed, thus making their comparison quite difficult [37]. However, a meta-analysis published the same year presented contradicting findings, as it highlighted that there was a significant advantage for ALC when compared to placebo for both clinical scales and psychometric tests; ALC also presented safe and tolerable long-lasting effects [38].

### 1.4. Aim And Rationale

Since 2003, other studies have been published, not only in AD but also in some secondary dementias and other cognitive disorders, including hepatic encephalopathy (HE). In particular, our research group has dealt with this topic, focusing on the possible cross-talk mechanisms of ALC within the gut-liver-brain axis. However, a critical review of the more recent trials and the mechanisms of action of ALC in dementia is still lacking. The purpose of this review is to provide an update on the existing literature dealing with ALC effects and tolerability in primary and secondary dementia. This in order to obtain a clear and comprehensive overview of all clinical benefits, potential side effects, limitations, and translational implications in the management of these patients.

## 2. Data Source and Selection

A Medline (PubMed)-based literature review was performed by using the following search terms, in different combinations: “acetyl-L-carnitine”, “dementia”, “neurodegeneration”, “cognitive disorder”, “cognitive impairment”, “cognitive decline”, “cognition”, “memory”, and “vascular”.

Two independent authors (V.P. and L.V.) screened all titles and abstracts of the retrieved publications, from the database inception to March 2020. Disagreements were solved by the consensus of a third author (F.F.) The studies had to include individuals with a clinical diagnosis of any type of dementia and severity according to the internationally accepted guidelines or diagnostic criteria. Of note, earlier trials were carried out when these guidelines were not in common use; in these cases, careful assessment of the inclusion/exclusion criteria was made, and trials were included if the criteria seemed likely to include people with dementia or cognitive impairment.

Duplicated entries, studies on physiological brain aging or diseases different from dementia or cognitive disorder, trials using ALC in nutraceutical formulation with other compounds, works on animals or cell cultures, studies not reporting the statistical analysis, non-English written papers, publications that are not research studies (i.e., commentaries, letters, editorials, reviews, etc.), conference, meeting proceedings, or any other paper not published in international peer-reviewed journals, study protocols, personal communications, or unpublished data, as well as any other study that did not fit with the scope of this review were excluded. Articles listed in the references were also reviewed in search for more data.

## 3. Results

**Figure 1:**
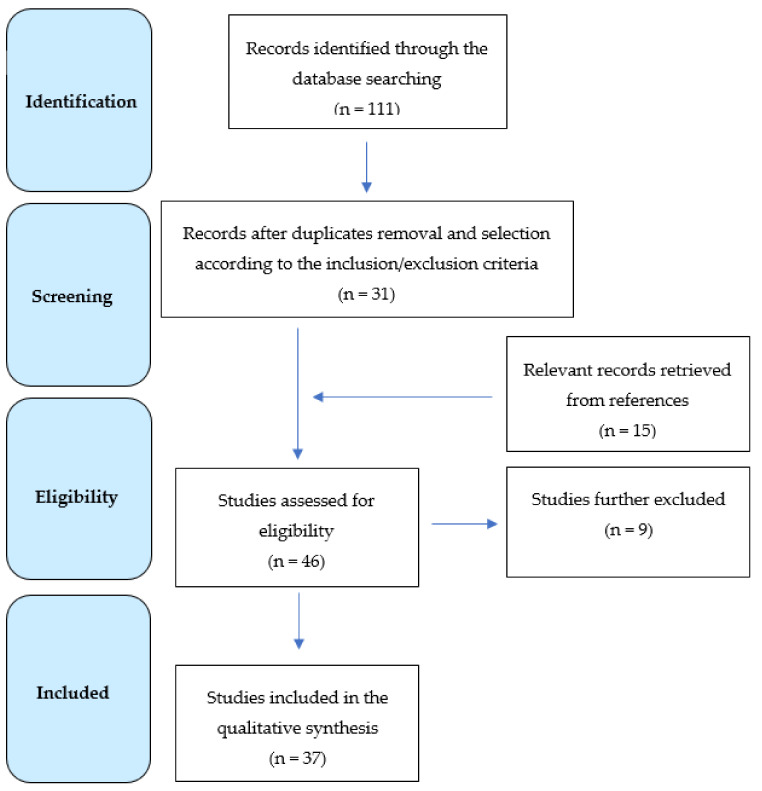
Flow diagram showing the search strategy, the number of records identified, and the included/excluded studies [71].

A total of 111 results were originally retrieved and screened. Of these, 31 publications were selected according to the inclusion and exclusion criteria. The examination of the references detected other 15 studies, whose analysis identified 6 additional papers fitting the purpose of this review. Therefore, a total of 37 papers were eventually included in the qualitative synthesis (Figure 1), and the main findings are summarized in Table 1. In more detail, nineteen studies deal with AD or MCI [16,20,23,29,36,39,40,41,42,43,44,45,46,47,48,49,50,51,52], three with vascular cognitive impairment (VCI) [53,54,55], five with HE [56,57,58,59,60], four with other secondary dementias [61,62,63,64], and six with an unspecified cognitive disorder [65,66,67,68,69,70].

### 3.1. Alzheimer’s Disease and Mild Cognitive Impairment

Previous articles highlighted that ALC supplementation was able to slower the progression in cognitive deterioration and behavioral disturbances of AD patients [16,23,29]. In two double-blind, randomized clinical trials in mild-to-moderate AD with a controlled placebo, the result of ALC 3 g/day for a year to the patients was a statistically significant slowed deterioration rate in various aspects of cognitive function and psychometric measures, with a good tolerability [16,49]. However, a later ad hoc designed study failed to confirm these evidences [50]. It is important to note that Spagnoli and colleagues recruited mild cases of AD [16], while in the study by Thal and collaborators in 1996, most of the individuals recruited were of moderate severity, with a baseline AD Assessment Scale Cognitive Subscale of 25.9 [49].

A different study found ALC to be efficacious after two-to-three months of treatment, which was an indication of the time required to modulate the cellular energy production [41]. In another trial whereby the dosage range used was between 1 and 2 g/day for 6–12 months, the result was an improvement, although not statistically significant, in several cognitive tests, such as word recognition, word list recall, and name learning, compared to placebo [44,72].

More objective results can be derived from the use of laboratory dosages or instrumental exams. A good example is highlighted by the increase in ALC plasma concentrations from 7.2 to 10.3 μM after 55 days of supplementation with 2 g/day [73]. The study done by Parnetti and colleagues found that the use of ALC IV or per os for 10–60 days led to increased ALC in the CSF of AD patients [20]. More recently, a single-photon emission computed tomography (SPECT) study that examined changes in cerebral perfusion relating to ALC administration in early AD patients found no changes in cognitive performance, dementia severity, and neuropsychiatric symptoms [52]. However, a significant increase in cerebral blood flow in the precuneus and a reduction of perfusion in the left inferior temporal gyrus, right insular cortex, and right middle frontal gyrus were reported [52]. The authors argued that, despite disease progression, cognitive performance did not aggravate and emphasized the role of precuneus in a variety of highly integrated functions, such as visuo-spatial imagery, retrieval of episodic memory, self-processing, and consciousness [74].

A meta-analysis of 21 studies conducted in patients with MCI to examine the effects of ALC against placebo found that it was effective in improving cognitive performance. Overall, the result depicted that 591 MCI patients treated with ALC in comparison to 613 placebo subjects had better scores for effect size measurements for both clinical and psychometric assessment scales and for the clinician’s assessment [38]. The highest efficacy was in memory and intellectual functions, that are the two most common and pronounced deficits observed in MCI subjects [38]. A narrow dose range of 1.5 to 2 g/day was used in the vast majority of patients; thus, the lack of significant dose-response is not surprising considering the complex ALC pharmacokinetic, that includes partial a prehepatic metabolism, a first pass liver effect, and varying bioavailability [38].

Regarding safety, the studies found that ALC had a high overall safety rating, and it was well tolerated without any significant side effects, even when administered for long periods (one year). The commonly reported adverse reactions included nausea, agitation, insomnia, and increased appetite [16]. The minimal side effects might also make ALC an alternative adjuvant treatment for patients with AChE inhibitors contraindication, intolerance, or adverse effects [35].

### 3.2. Vascular Cognitive Impairment

The term VCI is collectively employed to indicate the entire spectrum of vascular-related cognitive disorders, which range from a mild cognitive decline to an overt vascular dementia (VaD) [75,76]. The typical symptoms include executive dysfunction, mental slowness, memory deficit, behavioral disturbances, and depression, although they depend on the type, severity, extent, and location of the underlying cerebrovascular pathology [77,78].

The result of two double-blind placebo-controlled cross-over studies that tested ALC on the reaction time of post-stroke elderly subjects was that significant differences existed in memory, number, and word tests in the treated group, as well as in the responses to simple stimuli and the maze test performance [53,54]. Moreover, the authors did not report any side effect of ALC. In a very recent study [55], 56 patients with dementia and cerebrovascular disease participated in a multicenter, double-blind, placebo-controlled clinical trial and underwent randomized treatment comprising of ALC 1.5 g/day or placebo for 28 weeks. After ALC intake, the participants significantly improved in the score at the Montreal Cognitive Assessment, especially in the attention and language subitems. However, the possibility that the study purely dealt with VaD is hard to consider since patients were already on donepezil, which means that a not negligible proportion might have been affected by AD or mixed dementia [55].

### 3.3. Hepatic Encephalopathy

The evaluation of the effectiveness of ALC 4 g daily in HE has been done through four randomized controlled studies, with two of them focusing on minimal HE [57,58], another one in mild-to-moderate HE [59], and one in severe HE [60]. The overall finding is that treatment with ALC led to improved quality of life, anxiety, depression [58], and cognitive functions [57]. In more detail, subjects with mild and moderate HE significantly improved physical and mental fatigue, whereas severe HE subjects showed improvement in electroencephalography (EEG), cognitive and memory functions, visual scanning and tracking in computing ability.

In the two studies on populations with grade 4 of the West Haven HE rating criteria including severe HE and hepatic coma, respectively [60,79], the result was a decrease in serum ammonia and urea levels. Moreover, while serum ammonia and EEG improved in patients treated with ALC vs. placebo, the Glasgow Coma Scale (GCS) score worsened [79], whereas a different study in minimal HE found an improvement in GCS [57]. Drawing conclusions is hard because of low number of patients, difference of precipitating factors, and clinical heterogeneity.

A separate study highlighted that a single IV dose of ALC could improve the cerebral function of cirrhotic patients with HE as per the measurement of the P100 component of the visual evoked potentials [56]. The lack of any change in control subjects and the observation that the effect of ALC was rapid, transient, and not mediated by a reduction in plasma ammonia seem to indicate that ALC activity in HE is rather specific and probably directed to the central nervous system [56]. The findings highlighted are encouraging, but there is still need for more controlled studies on chronic ALC administration on both neuropsychological and electrophysiological tests that can help in the clinical application and outcome measures in HE of varying degree.

### 3.4. Other Secondary Dementias

Very few studies have assessed the effects of ALC in other secondary dementias.

Goetz and colleagues carried out a double-blind placebo-controlled crossover study to investigate ALC at the maximal permitted dose of 45 mg/kg/day for a week on 10 patients with Huntington’s disease [61]. The result was that ALC was neither efficient nor toxic, although some patients felt better after ALC, suggesting a subtle improvement that the assessment methods employed in this study could not detect [61]. Therefore, further replications are needed.

For patients with Human Immunodeficiency Virus infection, including those undergoing active antiretroviral therapy, the use of ALC supplementation has showed an anti-oxidant action able to lead to neuro- and cardio-protection, immunomodulation, and cognitive enhancement [80]. However, in Acquired Immunodeficiency Syndrome (AIDS)-dementia complex, only one study reported a significant improvement in motor and cognitive functioning after ALC IV [63].

The high prevalence of AD in patients with Down Syndrome led to the designing of a double-blind study that aimed at evaluating the impact of administering ALC 10–30 mg/kg/day to those patients for six months [64]. However, the detailed results were inconclusive [64].

In another study that saw 55 cognitively impaired patients with chronic alcoholism randomly assigned to either ALC 2 g/day or placebo, at baseline and after 45 and 90 days (T90), the finding was a significant difference in the T90 in favor of the ALC group [62]. The researchers employed Rey’s 15-word memory test, the Similarities WAIS subtest, the Wechsler memory scale, and the drawing coping test for understanding the differences.

### 3.5. Unspecified Cognitive Disorder

Older clinical studies defined subjects in different ways, such as “organic brain syndrome” [67], “geriatric patients with mental deterioration” [66]“, mild mental impairment” [65], “elderly with mental decline” [70], or “dementia” (not better specified) [68,69]. In contemporary studies, MCI acts as the best term for defining these patients.

Most of these studies found that ALC had positive cognitive and behavioral effects. Notably, Livingston and colleagues [69] did not find a statistically significant treatment effect for CGI, although the researchers included also patients with a quite high score of the Hachinski Ischemic Scale, which meant that also patients with VaD (especially those with multi-infarct dementia) might have been added in the analysis.

Another double-blind study with the placebo as the control that sought to investigate the ALC effect on the EEG of elderly outpatients with mild-to-moderate cognitive decline found a statistically significant effect both at 8 weeks (on the EEG) and 12 weeks of treatment (on the physician’s CGI and the patient-rated level of Activities of Daily Living) in favor of the treated group [67].

## 4. Discussion

### 4.1. Summary of the Evidences and Proposed Mechanisms

According to the studies reviewed here, ALC has been used for many years, and still continues to be used, as an adjuvant treatment for cognitive decline. Many of these trials were conducted several years ago; still, they were judged as reasonable considering that the placebo acted as the control in all of them, they were all randomized and double-blinded, and employed a parallel-group design. Nonetheless, although an evidence for benefit of ALC on CGI and MMSE at 24 weeks, an earlier Cochrane systematic review did not find the same results by using objective assessments in any area of outcome, thus concluding that there was insufficient evidence to recommend a routine use [37]. However, a meta-analysis of placebo-controlled studies presented contradictory findings as it found a significant clinical and psychometric advantage for ALC compared to placebo [38].

Overall, it is necessary to be cautious when interpreting these results, considering the potential influence that methodological difficulties and potential biases might have on them. Critics of metanalyses usually highlight bias and incomplete nature of selection, with a specific concern regarding the possibility of those studies with no effect being unpublished, which may provide misleading information since the published studies would not be representative of all the research done. The other problem is the differing nature of the methodologies employed by the studies: although they had similar design, route of administration, and indication, they had different test instruments, have been conducted in various settings, gave different daily doses of ALC, and had different treatment durations, which are all contributing factors to mixed results.

Interestingly, the findings of a recent study suggested a decrease of serum ALC and other acyl-carnitine concentrations along the continuum from healthy subjects to AD patients through subjective memory complaints and MCI, with acyl-carnitines metabolism being finely connected among them [81]. A suggestion made by the authors was that ALC serum level and that of other acyl-carnitines have the potential to identify patients before the conversion of the phenotype to AD, with the patient benefiting from the ALC treatment [81].

The restoration of cell membrane and synaptic function, enhancement of cholinergic activity, promotion of cellular energy, toxins protection, and neurotrophic exertion may be the neurobiological mechanisms underlying ALC intake in AD, MCI, and other degenerative disorders [15,82,83]. Indeed, many studies on ALC found that it exerts neuroprotective effects on oxidative stress and blocks neuronal death occurring in the pathophysiology of both brain aging and MCI [84].

Evidence highlighting the effects of ALC comes from animal studies, where ALC normalizes cerebral membrane alteration and metabolic energy during ischemia recovery [85,86]. Another finding is that ALC led to a prolonging of the survival of cultured rat brain cells after their exposure to neurotoxic stimuli [87] and to an improvement of cognitive functions [88]. Some studies also highlighted that oxidative mitochondrial decay is a contributing factor to aging and cognitive decline, and that feeding old rats with ALC could help in reversing some of these effects [89]. The proposal made by various researchers is that mitochondrial respiration is the main level for ALC action, which further leads to the production of adenosine triphosphate (ATP) that helps in the maintenance of a normal membrane potential [27,72,90]. Moreover, ALC regulates other intracellular pathways in rats, such as the protein kinase C [72,91]. Interestingly, a preclinical observation showed that ALC counteracted the loss of NMDA glutamate receptors in their neuronal membrane, as well as led to an increased production of neurotrophins, with the two effects relating to adaptive synaptic plasticity [72,92,93,94].

In AD models, ALC has proved to reduce the β-amyloid toxicity in rat’s primary cortical neurons through the activation of the heat shock protein (Hsp) HO-1 and the Hsp70 expression [95]. ALC was indeed effective in preventing age-related changes relating to mitochondrial respiration by modulating the Hsp family and by decreasing oxidative stress biomarkers [96]. Other aspects relating to ALC include life-span increment, improvement in behavior, and guaranteed long-term memory performance, as seen in aged rats [72]. Taken together, these beneficial effects, as well as those observed on fat metabolism and immune function, may be transferrable to dementia patients.

However, human brain responses to ALC still remain rather unknown. In one study employing ^31^P-magnetic resonance spectroscopy in AD patients, the phosphomonoester levels decreased after ALC treatment, while there was a normalization of high-energy phosphate at baseline, with these changes being evident only in the dorsal prefrontal area [23]. Further, in vivo imaging studies in mild AD patients revealed a decrease in the cholinergic activity in brain areas typically affected by AD, such as the precuneus and the posterior cingulate cortex [97,98], especially in very early stages [99]. Interestingly, although previous evidence has suggested that AD patients generally demonstrate progressive deterioration in brain perfusion and clinical symptoms, a recent SPECT study depicted an increase in the perfusion of the precuneus in AD patients after the administration of ALC, along with a lack of further deterioration of cognitive and neuropsychiatric symptoms [52]. In chronic alcoholics, the conclusion arrived at by the authors was that ALC has great efficacy and safety score concerning cognitive disturbances, as well as the capability of improving all cognitive areas explored, possibly acting either at the cholinergic transmission or at the neuronal metabolism level [62].

A cholinergic impairment seems to play a role also in VCI pathophysiology and neurochemistry. The core imaging findings of these patients are focal, multifocal, or diffuse ischemic lesions involving various cortical and subcortical regions, with consequent deafferentation of frontal and limbic cortical areas and interruption of basal ganglia, thalamus, and sub-frontal areas [100,101,102]. Dense networks of cholinergic fibers within the injured area are thought to be responsible for cholinergic dysfunction [103,104] and for the positive effects that AChE inhibitors exert in some patients with VaD [105]. Another finding from models of experimental ischemia was the delay in brain damage resulting from post-ischemic inflammation [106]. In this scenario, a recent review underlined that ALC could improve tolerance to ischemia and reperfusion injury [107].

Finally, patients with AIDS-dementia complex exhibited clinical improvements that were parallel to a significant reduction of glutamate concentration in both CSF and serum [63]. A prospective study on the effect of ALC in AIDS patients found that it could reduce blood glutamate level in order to prevent the neurotoxicity by nucleoside analogues exposure. The authors conclude that ALC can act as an anti-apoptotic agent, even if there are still no established mechanisms accounting for the observed reduction of glutamate [63].

### 4.2. Potential Organ Cross-Talk Actions of ALC and Hepatic Detoxifying Mechanisms

The novel insights on the effects of ALC in HE contribute to the further understanding of the mechanisms of action of ALC, which may include the organ cross-talk and the improvement of hepatic detoxifying mechanisms.

From the studies focusing on HE, most of them carried out by our research group, it was noted that ALC may act on various levels, with its administration at a supraphysiological concentration having the capacity of reducing serum ammonia and exerting protective effects against glutamate and ammonium neurotoxicity [108]. The excess of extracellular glutamate under ammonium exposure is excitotoxic through the activation of NMDA receptors and leads to alteration in nitric oxide metabolism, disturbances in Na^+^/K^+^ ATPase, ATP shortage mitochondrial disfunctions, free radical accumulation, and oxidative stress [109]. The glutamatergic excitotoxicity under ammonium exposure can also alter other neurotransmission systems, such as the γ-amino-butyric acid or the activity of benzodiazepine receptors [110,111]. Consequently, it has hypothesized that ALC may have a dual protective effect on the cell, i.e., by enhancing the energy dynamics and by inhibiting membrane hyperexcitability. Excitotoxic damage via the upregulation of NMDA receptors highly depends on the cellular energy state, with ALC inducing ureagenesis that causes a decrease in blood and brain level of ammonia as well [79].

Our studies suggest new insights that might contribute to the clinical effects observed in some cognitive and behavioral symptoms of HE, such as irritability, sleep disorders, psychomotor retardation, and thinking disturbances, which all negatively impact quality of life and healthcare expenses. As known, ALC is an endogenous compound that acts as a donor of acetyl groups and facilitates the transfer of fatty acids from cytosol to mitochondria during beta-oxidation. The gut–liver axis plays a relevant role in the oral absorption of ALC, which is deacetylated immediately after its uptake into intestinal cells, with a portion of the newly formed intracellular free carnitine being re-acetylated [19]. Given that the conversion of ALC to carnitine is higher than the average renal clearance of ALC, a large proportion of the acetyl moieties is used for biosynthetic pathways. In particular, ALC provides the acetyl-CoA group for the synthesis of acetylcholine in the brain and fatty acids in lipogenic tissue (both containing the fatty acids synthases, as well as the liver) [15].

In cellular cultures, the ALC-induced decrease in oxidative stress occurred not only by exerting a protective effect on mitochondrial structure and functioning, thus making the electron transport chain less prone to electron leak and superoxide production, but also by stimulating the mechanisms of endogenous cellular antioxidants defense [112]. While medium- and short-chain fatty acids can freely enter the mitochondrion through a membrane diffusion, ALC is needed for the mitochondrial transfer of activated long-chain fatty acids, a series of reactions globally called as the “carnitine shuttle” [82,113]. This system is crucial to prevent the accumulation of long-chain fatty acids and long-chain acyl-CoA, which can be deleterious to the cells [114].

Carnitine also stimulates the mitochondrial respiratory rate and influences the properties of the mitochondrial membranes. Therefore, a reduction of carnitine may cause a decreased availability of long-chain fatty acids in the mitochondria and, more generally, an energetic deficit in the most energy-demanding tissues, such as the skeletal muscle [115]. Since ALC is metabolized to acetyl-CoA, it has the potential to acetylate the histones (which modulate the gene expression) and proteins (such as enzymes and related activities) [116,117,118,119]. Hence, an increase in metabolic rate without a parallel increase of electron quenching mechanisms in the target tissues or in sensitive cells (such as neurons) may be required to prevent detrimental effects. These organ cross-talk mechanisms and the indirect effects of ALC via the improvement of liver and skeletal muscle metabolism, and thus the overall metabolism, might also explain the differences observed among trials and help the design of future studies by using objective measures of outcome and mechanisms of action of ALC.

### 4.3. Current Limitations and Research Agenda

Although the findings highlighted here seem to be quite encouraging, it is crucial to consider some limitations and critical aspects. First, the period covered by the studies is more than thirty years, a period which has seen a considerable change in the diagnostic criteria, a variation in the test measures used, and a significant development of the methodology for AD and MCI investigation. Future studies need also to include individual patient data and to consider a broader range of outcome measures, like the mood status and the caregiver’s quality of life.

More research on the pharmacodynamics of ALC in humans is also needed. Even with a favorable pharmacokinetic profile (such as the ability to cross the BBB), there is a trend of ascribing the lack of effect of ALC in AD to its pharmacodynamics [120]. Such findings have led to the consideration of administering natural substances at low doses as part of a new drug-delivery system to improve bioavailability and brain penetrance. The intriguing aspect is that this can help to minimize adverse effects developing from unwanted fluctuations in plasma concentrations.

Finally, it is crucial to consider the warning represented by the patient’s educational level, which differs across the studies. The effects of education on cognitive status assessment are well known [121]. However, as evidenced by previous studies, a correlation between all outcome measures and educational level did not occur, mainly because such an influence would take place in both treated patients and placebo group, which means the difference would be eliminated during the effect sizes calculation [38]. Nonetheless, larger samples matched for age, sex, and education are warranted.

## 5. Conclusions

Based on the currently available evidences, the role of ALC in AD and other cognitive disorders is still under debate. Future multicenter double-blind, randomized, placebo-controlled trials in a large and homogeneous sample of patients should focus on higher doses and more prolonged treatment. Longitudinal studies with multidimensional assessments and a wide range of outcome measures are also needed before a systematic application of ALC in clinical practice. In this scenario, our research group will continue to deal with the role of ALC in HE of varying severity, with the translational goal to not only test the clinical effects but also to further understand the complex but fascinating interactions within the gut-liver-brain axis.

## Figures and Tables

**Table 1:** Studies on acetyl-L-carnitine (ALC) in dementia and other cognitive disorders.

Disorder	Reference	Study Characteristics	Main Results
**Alzheimer’s Disease/Mild Cognitive Impairment**	Battistin et al., 1989 [39]	Type of study: randomized, double blind placebo controlled Subjects: 137 AD patients Treatment: ALC 2 g die orally for 180 days Outcome measures: Digit span, block tapping, verbal fluency, copying test, MMSE, BDS, BIMC, Raven’s matrices, digit symbol, Rey’s test	Improvement in verbal fluency in the treated group.
	Battistin et al., 1989 [40]	Type of study: prospective Subjects: 30 demented patients (21 AD, 9 with mixed dementia) randomly assigned to different groups Treatment: ALC 0.5 g, 1 g, 1.5 g, or 2 g die IV Outcome measures: regional cerebral blood flow measured with SPECT with 99mTc-HM-PAO	The lowest dose of ALC did not produce changes. Uptake increases in the parietal cortex after higher doses intake; significant elevations in the frontal cortex after 1.5 g and in the temporal region after 2 g. When side-to-side cortical asymmetries were present, they significantly reduced after ALC. 99mTc-HM-PAO uptake increased in thalamus after 1 and 1.5 g.
	Bellagamba et al., 1990 [41]	Type of study: randomized, double-blind, parallel, placebo controlled Subjects: 35 AD patients randomized in real (16) and placebo (19) groups Treatment: ALC 3 g die orally for 3 months Outcome measures: SCAG, Rey’s Auditory Verbal Learning Test, Raven PM 47 and TP Barrage Test, CGI	Significant improvement in behavioral profile, memory, attention, intellective function, and CGI for ALC compared to placebo.
	Campi et al., 1990 [42]	Type of study: single-blind, randomized, parallel Subjects: 40 AD patients randomized in ALC and selegiline groups Treatment: ALC 0.5 g bis in die orally for 90 days Outcome measures: psychometric examination, at baseline and every 30 days	Selegiline led to a global improvement in processing, storage, retrieval of given information, verbal fluency, and visuospatial abilities. Excellent tolerability of both drugs.
	Passeri et al., 1990 [43]	Type of study: randomized, double-blind, placebo controlled Subjects: 60 MCI patients randomized in real (30) and placebo (30) groups Treatment: ALC 2 g die orally for 3 months Outcome measures: MMSE, SHGRS, BDS, BIMC, Rey’s test, Corsi’s test, verbal fluency, TP, digit span, HDRS, Gibson’s test	Statistically significant improvement in behavioral scales, memory tests, attention barrage test, and verbal fluency test for ALC compared to placebo.
	Rai et al., 1990 [44]	Type of study: randomized, double-blind, parallel, placebo controlled Subjects: 20 AD patients randomized in real (7) and placebo (13) groups Treatment: ALC 1 g bis in die orally for 24 weeks Outcome measures: P300 latency of the auditory evoked potentials, GDS - short form; Kendrick Battery Tests, OLT and Digit Copying Test, NLT, Word Fluency Test-modification of Set Test, ADL	No changes for P300 latency, ADL, GDS, Digit Copying Test and Word Fluency Test. Trend for more improvement in relation to the NLT, computerized Digit Recall Test and reaction time in the ALC group compared to placebo.
	Spagnoli et al., 1991 [16]	Type of study: randomized, double-blind, parallel, placebo controlled Subjects: 130 AD patients Treatment: ALC 2 g die orally for one year Outcome measures: SBI, BDS, BIMC, Raven’s matrices, prose memory, apraxia, finger agnosia	Compared to controls, significant improvement in BDS, ideomotor and bucco-facial apraxia, logical intelligence, and selective attention for ALC. Better performance at BDS, logical intelligence, verbal critical abilities, long-term verbal memory, and selective attention at the analysis of covariance.
	Parnetti et al., 1992 [20]	Type of study: prospective Subjects: 11 AD patients Treatment: ALC 30 mg/kg bis in die IV for 10 days; ALC 2 g die orally in 3 daily doses for 50 days Outcome measures: ALC concentration in plasma and CSF	IV and oral administration of multiple doses of ALC increased both plasma and CSF concentration of ALC.
	Sano et al., 1992 [29]	Type of study: randomized, double-blind, parallel, placebo controlled Subjects: 27 AD patients randomized in real (13) and placebo (14) groups Treatment: ALC 2.5 g die for 3 months, followed by 3 g die for 3 months Outcome measures: SRT; modified MMS, which included: Digit Span (forward-backward); the Logical Memory, Paired Associate, and Visual Reproduction subtests from the Wechsler Memory Scale; the Benton Visual Retention Test-Multiple Choice Version; verbal fluency test for letter; the category naming test; cancellation test scoring time and errors; SIP, SMQ, CGI; CSF ALC	Significantly less deterioration in timed cancellation tasks and Digit Span (forward) and a trend toward less deterioration in a timed verbal fluency task for ALC group compared to placebo; no difference in any other neuropsychological test. A subgroup with the lowest baseline scores had significantly less deterioration on the verbal memory test and a significant increase in CSF ALC levels compared to placebo.
	Costa et al., 1993 [45]	Type of study: randomized, parallel, double blind, placebo controlled Treatment: ALC 2 g die for 16 weeks Outcome measures: Reys test, Digit span, Corsi’s test, digit symbol test, Gibson’s spiral, verbal fluency, GBS	ALC normalized cortisol and ACTH level in response to a CRF stimulation test and reduced the number of non-suppressants in a dexamethasone test. Improvement at Digit Symbol Substitution test, verbal fluency test, and Rey’s verbal test.
	Bayer 1994 [46]	Type of study: randomized, double blind, placebo controlled Subjects: 30 AD patients Treatment: ALC 2 g die for 24 weeks Outcome measures: MMSE, HRSD, BDS, GBS, KOLT, KDCT, CGI-S, CGI-C, CGI-E, Relatives Assessment	Improvement in CGI for the treatment group compared to placebo.
	Mullin 1994 [47]	Type of study: randomized, parallel, double blind, placebo controlled Subjects: 62 AD patients Treatment: ALC 2 g die orally for 180 days Outcome measures: MMSE, CGI, GDS, NART, KOLT, KDGT, NLT, ADL, Word Fluency	No significant difference between treatment and control group.
	Bruno et al., 1995 [48]	Type of study: open label Subjects: AD patients Treatment: ALC at highly dose IV Outcome measures: CSF and plasma ALC concentrations	CSF levels under ALC treatment were significantly higher compare to baseline. Beta-endorphins significantly decreased after treatment, with plasma cortisol levels matching this reduction.
	Pettegrew et al., 1995 [23]	Type of study: double-blind, placebo controlled Subjects: 12 AD patients randomized in real (7) and placebo (5) groups Treatment: ALC 3 g die orally for one year Outcome measures: MMS, ADAS, membrane phospholipid and high-energy phosphate metabolism measured by ^31^P-MRS	Patients showed significantly less deterioration in MMS and ADAS scores. The decrease in phosphomonoester and high-energy phosphate levels observed in both ALC and placebo groups at baseline was normalized in the real group but not in the placebo group.
	Thal et al., 1996 [49]	Type of study: randomized, double-blind, parallel, placebo controlled Subjects: 419 AD patients randomized in real (207) and placebo (212) groups Treatment: ALC 3 g die orally for one year Outcome measures: primary: ADAS, CDR; secondary: ADAS-non-cognitive subscale, MMSE, ADL, IADL, CGI-S and CGI-C	Both groups declined on primary and most of secondary measures during the trial. A trend for early-onset patients on ALC to decline more slowly than placebo on both primary endpoints was found; conversely, late-onset AD patients on ALC tended to progress more rapidly than early-onset.
	Brooks et al., 1998 [36]	Type of study: randomized, double-blind, parallel, placebo controlled Subjects: 334 AD patients randomized in real (165) and placebo (169) groups Treatment: ALC 3 g die orally for one year Outcome measures: primary: ADAS, CDR; secondary: ADAS, CDR; secondary: ADAS-non-cognitive subscale, MMSE, ADL, IADL, CGI-S and CGI-C	Reanalysis of the data by Thal et al., 1996 by using the trilinear approach, in which measurements are allowed to follow a pattern of stability-change-stability. Both groups exhibited the same mean rate of change on ADAS. Multiple regression analysis revealed younger subjects benefiting more from ALC.
	Thal et al., 2000 [50]	Type of study: randomized, double-blind, placebo controlled Subjects: 229 AD patients randomized in real (112) and placebo (117) groups Treatment: ALC 1 g ter in die orally for one year Outcome measures: primary: ADAS, CDR; secondary: ADAS-non-cognitive subscale, MMSE, ADL, CIBIC	No significant difference was found in the primary outcomes; less deterioration in MMSE score was observed for ALC, whereas no difference for ADL and CIBIC.
	Bianchetti et al., 2003 [51]	Type of study: open-label Subjects: 21 AD patients treated with donezepil or rivastigmine. Treatment: ALC 2 g die orally for 3 months Outcome measures: ADAS-cognitive subscale, MMSE	Response rate (defined as a reduction of ADAS-Cog score ≥4) increased from 38% to 50% after ALC administration.
	Jeong et al., 2017 [52]	Type of study: prospective Subjects: 18 AD patients Treatment: ALC 1.5 g die orally for 1.4 ± 0.3 years Outcome measures: SPECT with ^99^mTc-HM-PAO, MMSE, CDR, GDS, NPI	Non-significant changes in MMSE, CDR, GDS, and NPI. Cerebral perfusion significantly increased in the right precuneus, whereas it reduced in the left inferior temporal gyrus, the right middle frontal gyrus, and the right insular cortex
**Vascular Cognitive Impairment**	Arrigo et al., 1988 [53]	Type of study: randomized, double-blind, crossover Subjects: 12 patients with cerebrovascular insufficiency (not further specified) Treatment: ALC 1.5 g die orally for 4 weeks Outcome measures: verbal and numerical memory, non-verbal performance, reaction times (simple and multiple-choice) and EEG	ALC significantly more active than placebo in memory and non-verbal tests and in simple reaction times.
	Arrigo et al., 1990 [54]	Type of study: double-blind, cross-over Subjects: 12 elderly patients with acute brain circulatory insufficiency Treatment: ALC 1.5 g die orally for 4 weeks Outcome measures: memory, number, and word tests; responses to simple stimuli and performance of the maze test	Significant differences between drug and placebo in memory, number, and word tests, as well as in the responses to simple stimuli and the performance of the maze test. No side effects.
	Yang et al., 2018 [55]	Type of study: multicenter, randomized, double-blind, placebo-controlled, parallel-group trial Subjects: 56 patients with dementia and cerebrovascular disease, randomized in real (30) and placebo (26) groups Treatment: ALC 0.5 g ter in die for 28 weeks Outcome measures: primary measure: MoCA-K; secondary measures: K-MMSE, K-CWST, COWAT, K-TMT-E, K-IADL, GDS, CDR, CDR-SB	Cognitive function measured by the MoCA-K significantly improved in the ALC-treated group, in particular the attention and language sub-items. No difference in secondary outcome measures.
**Hepatic Encephalopathy**	Siciliano et al., 2006 [56]	Type of study: prospective Subjects: 18 cirrhotic patients with HE and 6 with previous TIA as controls Treatment: ALC 0.5 g in 50 mL isotonic saline IV (infusion rate 10 mL/min) Outcome measures: pattern reversal P100 latency of visual-evoked potentials	Significant reduction in P100 latencies 30 min after ALC infusion in HE patients. The mean P100 latencies measured in HE subjects was significantly shorter after ALC infusion compared with values obtained before ALC administration.
	Malaguarnera et al. 2008 [57]	Type of study: randomized, double-blind, placebo controlled Subjects: 115 minimal HE patients randomized in real (60) and placebo (55) Treatment: ALC 2 g bis in die orally for 90 days Outcome measures: TMT, BDT, AVL, MMSE, and EEG	Improvement of TMT-A, TMT-B, MMSE, BDT, SDMT, and AVL scores in the real group; no difference in EEG. No differences in the treated group compared to baseline and placebo in both neurophysiological and neuropsychological assessment.
	Malaguarnera et al., 2011 [58]	Type of study: randomized, double-blind, placebo controlled. Subjects: 67 minimal HE patients randomized in real (33) and placebo (34) Treatment: ALC 2 g bis in die orally for 90 days Outcome measures: Line Drawing Test, Serial Dotting Test, TMT, SF-36, BDI, STAI, and EEG	Significant improvements in BDI, TMT-B, STAI, and line tracing in real group compared to baseline and placebo group. No differences in EEG.
	Malaguarnera et al., 2011 [59]	Type of study: randomized, double-blind, placebo controlled Subjects: 121 HE patients divided in mild (HE1: 61) and moderate (HE2: 60), randomized to ALC (31 HE1 and 30 HE2) or placebo (30 HE1 and 30 HE2) Treatment: 2 g ALC bis in die orally for 90 days Outcome measures: FSS, Wessely’s test, Powell’s test, 7-d PAR, SPPB, 6-min walking test, and EEG.	The ALC-treated patients in the HE1 group showed significant improvement than placebo group in mental fatigue score, FSS, 7-d PAR score, and SPPB. The HE2 group showed significant improvement in FSS and in the 6-min walking test.
	Malaguarnera et al., 2011 [60]	Type of study: randomized, double-blind, placebo controlled Subjects: 60 severe HE patients randomized in real (30) and placebo (30) groups Treatment: ALC 2 g bis in die orally for 90 days Outcome measures: TMT, digit cancellation, MMSE, COWAT, JLO, Logical Memory (Paragraph Recall), EMQ, Hooper visual organization test, and EEG	Significant improvement in the real group compared to placebo for EMQ, Paragraph Recall, TMT-A, TMT-B, COWAT, Hooper test, JLO, and digit cancellation time.
**Others Secondary Dementias**	Goetz et al., 1990 [61]	Type of study: randomized, double-blind, placebo controlled, crossover Subjects: 10 patients with HD randomized to real and placebo treatment and switched over after a 2-week drug-free period. Treatment: ALC 45 mg/kg/day for one week Outcome measures: Shoulson–Fahn Disability Scale for HD, AIMS, reaction time, MMSE, HDRS, Verbal Fluency using the Controlled Oral Word Association Test, global severity assessment from AIMS.	Both placebo and ALC significantly improved reaction time compared to baseline and did not significantly differed from each other. No serious side effects of ALC during the study.
	Tempesta et al., 1990 [62]	Type of study: randomized, double-blind, placebo controlled Subjects: 55 chronic alcoholic patients randomized in real and placebo group Treatment: ALC 2 g die orally for 90 days Outcome measures: memory, constructional praxia, deductive-logical functions, and language. Testing time at baseline (T0), after 45 (T45) and 90 (T90) days	At T90, significant differences favoring the real treatment on the Rey’s 15-word memory test, the Wechsler memory scale, and the Similarities WAIS subtest were noted. On the copying drawing test, the placebo group did not show any T0-T90 variation, while improvement was observed in the ALC group.
	Famularo et al., 1999 [63]	Type of study: case report, followed by a prospective pilot study Subjects: one patient with AIDS-dementia complex Treatment: single course of ALC IV Outcome measures: motor and cognitive tests; blood and CSF glutamate level	Significant motor and cognitive improvement after ALC, along with a significant reduction of glutamate concentration in both blood and CSF, as confirmed by the prospective pilot study on blood levels of glutamate in AIDS patients.
	Pueschel, et al., 2006 [64]	Type of study: randomized, double-blind, placebo controlled Subjects: 40 Down syndrome randomized in real and placebo group Treatment: ALC 10 mg/kg/day (first month), 20 mg/kg/day (second month), 30 mg/kg/day (next 4 months) Outcome measures: SBIS, Hiskey–Nebraska VAS, MFFT, WISC-r, KABC, VABS, CBCL; assessment performed at follow-up of 3, 6, and 9 months	No significant difference between the two groups.
**Unspecified cognitive disorder**	Passeri et al., 1988 [65]	Type of study: randomized, double-blind, placebo controlled Subjects: 30 elderly patients with “mild mental impairment” randomized in real and placebo group (15 patients each) Treatment: ALC 2 g die orally for 3 months Outcome measures: BDS, SHGRS, Rey short-term, Rey long-term, Corsi, Barrage, and Verbal Fluency	Overall, no significant difference between groups. ALC treated patients showed improvement in BSD, SHGRS, Rey short- and long-term memory tests, Corsi, Barrage test, and in the Verbal Fluency test.
	Mantero et al., 1989 [66]	Type of study: double-blind, placebo controlled Subjects: 50 patients with dementia (not further specified) Treatment: 2 g ALC die orally for 180 days Outcome measures: MMSE, BDS, BIMC, HRSD, CGI-C, CGI-S, CGI-E, HIS	Improvement in MMSE, CGI, and BDS in the treated group.
	Herrmann et al., 1990 [67]	Type of study: randomized, double-blind, placebo controlled Subjects: 230 patients aged 60–80 with cognitive decline corresponding to stages 3 or 4 on GDS (80% with stage 3), randomized to real and placebo group Treatment: ALC 1.5 g die for 3 months Outcome measures: CGI-C, NGDAS, modified digit symbol substitution test	Significant effect of the ALC treatment on the physician’s CGI and the patient-rated level of ADL.
	Sinforiani et al., 1990 [68]	Type of study: single-blind Subjects: 24 patients with mild-to-moderate dementia according to DSM III criteria divided in ALC group and piracetam group (12 patients each) Treatment: ALC IV for 2 weeks followed by oral ALC for 10 weeks Outcome measures: cognitive, attentive, and behavioral functions	Statistically significant improvement in behavioral, attentional, and psychomotricity features of patients treated with ALC.
	Livingston et al., 1991 [69]	Type of study: randomized, double-blind, parallel, placebo controlled Subjects: 71 patients with dementia randomized in real (35) and placebo (36) Treatment: ALC for 24 weeks (dosage not specified) Outcome measures: MMSE, KOLT, word fluency, drawing, recognition memory for words and pictures, modified NLT, PADL, GCI	Statistically significant improvement in the recognition memory in the ALC group.
	Salvioli and Neri 1994 [70]	Type of study: single-blind Subjects: 481 elderly patients with “mental decline” (not further specified) Treatment: phase T0: placebo treatment for 30 days; T1 and T2: ALC 1.5 g die orally for 90 days; T3: further 30 days of placebo treatment Outcome measures: MMSE, Randt Memory Test, GDS, HDRS, FSS	Significant improvement observed during and after ALC treatment.

Legend (in alphabetic order): 7-d PAR: 7-d Physical Activity Recall questionnaire; AD: Alzheimer disease; ADAS: AD assessment scale; ADL: activities of daily living; AIDS: acquired immunodeficiency syndrome; AIMS: Abnormal Involuntary Movement Scale; ALC: acetyl-L-carnitine; AVL: Auditory Verbal Learning Test; BDI: Beck depression inventory; BDS: Blessed Dementia Scale; BDT: block design test; BIMC: Blessed Information Memory and Concentration Test; CBCL: Child Behavior Checklist; CDR: clinical dementia rating scale; CDR-SB: Clinical Dementia Rating Scale Sum of Boxes; CGI: clinical global impression; CGI-C: clinical global impression of change/improvement; CGI-E: clinical global impression of efficacy; CGI-S: clinical global impression of severity; CIBIC: Clinician-Based Impression of Change; COWAT: Controlled Oral Word Association Test; CRF: corticotropin-releasing factor; CSF: cerebrospinal fluid; EMQ: Everyday Memory Questionnaire; FSS: Fatigue Severity Scale; GBS: Gottfries–Brane–Steen Scale; GDS: geriatric depression scale; HD: Huntington disease; HDRS: Hamilton depression rating scale; HE: hepatic encephalopathy; HIS: Hachinski Ischaemic Score; HM-PAO: hexamethylpropyleneamine oxime; IADL: instrumental activities of daily living; IV: intravenously; JLO: Judgement of line orientation; K-CWST: Korean-Color Word Stroop Test; KDCT: Kendrick Digit Copying Test; K-IADL: Korean Instrumental Activity of Daily Living; KABC: Kaufman Assessment Battery for Children; K-MMSE: Korean MMSE; KOLT: Kendrick Object Learning Test; K-TMT-E: Korean-Trail Making Test-Elderly’s Version; MCI: mild cognitive impairment; MFFT: Matching Familiar Figure test; MMS: Mini-Mental Status; MMSE: Mini Mental State Examination; MoCA-K: Korean version of Montreal Cognitive Assessment; MRS: magnetic resonance spectroscopy; NART: National Adult Reading Test; NAS: Nuremberg Gerontopsychological Self-Rating Scale for Activities of daily Living; NGDAS: Nuremberg Geriatric Daily Activities Scale; NLT: name learning test; NPI: neuropsychiatric inventory; OLT: object learning test; PADL: performance of activities of daily living; RGDS: Reisberg Global Deterioration Scale; SBI: spontaneous behavior interview; SCAG: Sandoz Clinical Assessment Geriatric; SDMT: Symbol Digit Modalities Test; SF-36: 36-item short-form; SHGRS: Stuard Hospital geriatric rating scale; SBIS: Stanford–Binet Intelligence Scale; SIP: Sickness Impact Profile; SMQ: Squire’s Memory Questionnaire; SPPB: Short Physical Performance Battery; SRT: Verbal Selective Re-minding Test; STAI: State-trait anxiety inventory; TMT: trail making test; TP: Tolouse–Pieron; VABS: Vineland Adaptive Behavior Scale; VAS: Visual Attention Span; VCI: vascular cognitive impairment; WAIS: Wechsler Adult Intelligence Scale; WISC-r: Wechsler Intelligence Scale for Children-Revised; words in bold: articles not discussed in the 2003 Cochrane systematic review [37].
